# Active8: a randomized, double-blind, placebo-controlled study of chemotherapy plus cetuximab in combination with TLR8 agonist VTX-2337 in patients with recurrent or metastatic squamous cell carcinoma of the head and neck (SCCHN)

**DOI:** 10.1186/2051-1426-2-S3-P69

**Published:** 2014-11-06

**Authors:** Robert L Ferris, Ezra Cohen, Kelly Gash, Sam Whiting, Kristi Manjarrez, Greg Dietsch, Robert Hershberg, James Kyle Bryan

**Affiliations:** 1 University of Pittsburgh Cancer Institute, Pittsburgh, PA, USA; 2 UC San Diego Health System - La Jolla, La Jolla, Ca, USA; 3 VentiRx Pharmaceuticals, Seattle, WA, USA

## Background

Recurrent or metastatic SCCHN has few effective therapeutic options. In these patients, the EXTREME regimen added Cetuximab--an EGFR-specific monoclonal antibody--to a regimen of Platinum/5-FU and improved median overall survival (OS) by 2.7 months and progression-free survival (PFS) by 2.3 months [1]. Given the natural killer (NK) cell-mediated antibody dependent cellular cytotoxicity (ADCC) of Cetuximab-mediated tumor killing and the immunogenic antitumor effects of chemotherapeutics like Platinum and 5-FU, synergy with immunotherapy may be one option to further improve outcomes.

VTX-2337 (Motolimod), a novel Toll-like receptor 8 (TLR8) agonist, stimulates myeloid dendritic cells (mDC), monocytes, and NK cells. Preclinical data have demonstrated increased ADCC with IgG1 monoclonal antibodies when Motolimod is added [2] as well as synergistic effects when Motolimod is combined with chemotherapy [3]. A Phase Ib study of Cetuximab and Motolimod in patients with Platinum-refractory or -intolerant recurrent or metastatic SCCHN has shown the combination to be both tolerable and active in this setting [4]. Translational medicine correlates demonstrated TLR8 activation and enhanced NK cell mobilization and activation. There is strong rationale to further assess the safety, tolerability, and efficacy of this combination in SCCHN patients. A large trial in this population will allow exploration of a number of translational medicine correlates to illuminate the combination of immunotherapy with other anti-cancer therapies.

## Methods

This randomized, double-blind, placebo-controlled, Phase II study evaluates the safety and efficacy of Motolimod with EXTREME vs. EXTREME alone for first-line treatment of recurrent or metastatic SCCHN. Patients must have a confirmed diagnosis, measurable disease, and no prior systemic therapy for their recurrent or metastatic disease. Approximately 175 patients will be randomized 1:1 to receive Motolimod or placebo + EXTREME for 6 cycles (q3wk), followed by weekly Cetuximab with biweekly Motolimod/placebo. The primary objective is to compare PFS according to immune-related response evaluation criteria between the two treatment arms, as determined by independent review. Secondary objectives include comparisons of safety and OS between the two treatment groups.

Translational studies will be conducted to compare immune biomarker response by a multiplexed panel of cytokines, chemokines, and inflammatory markers; genetic polymorphisms that may impact response to a TLR8 agonist or to Cetuximab; and the effect of immune cell subsets within the tumor on response to treatment and/or clinical outcome between the two treatment groups. Pharmacokinetics of Motolimod will also be evaluated. Enrollment began in October 2013. (NCT01836029)
